# Cardiovascular disease prevention should start in early life

**DOI:** 10.1186/s44263-023-00015-4

**Published:** 2023-09-07

**Authors:** Jean Jacques Noubiap, Ulrich Flore Nyaga

**Affiliations:** 1 Division of Cardiology, Department of Medicine, University of California-San Francisco, San Francisco, CA USA; 2 Department of Medicine and Specialties, Faculty of Medicine and Biomedical Sciences, University of Yaoundé I, Yaoundé, Cameroon

**Keywords:** Cardiovascular disease, Prevention, Physical activity, Hypertension, Obesity, Adolescents, Young adults

## Abstract

Adolescence and young adulthood are critical periods for building the foundation of cardiovascular health. Unfortunately, the incidence of cardiovascular disease has substantially increased in adolescents and young adults in the last three decades. Multilevel interventions are needed to preserve ideal cardiovascular health in this population.

## Background

Cardiovascular diseases (CVD) continue to inflict a colossal health and economic burden to all societies. It remains the leading cause of mortality and disabilities globally, with a substantial increase in prevalent cases (271 million to 523 million), disability-adjusted life years (DALYs, 279.8 million to 393.1 million), years lived with disability (17.7 million to 34.4 million), and deaths (12.1 million to 18.6 million) between 1990 and 2019 [1]. Low- and middle-income countries are disproportionally affected, accounting for over 80% of global CVD cases and related deaths [1]. Although a decline in age-standardized mortality for specific CVD such as ischemic heart disease occurred in several high-income countries in the previous decades, a rebound in mortality has been observed over the past 5 years in countries like the USA and UK [1]. These disturbing trends call for improved cardiovascular prevention and health care in all parts of the globe.

Because CVD, especially atherosclerotic CVD, mostly occurs after the third decade of life, it has long been overlooked in adolescents and young adults. While most adolescents do not have CVD, many of them have CVD risk factors, especially reduced physical activity and poor diet [2]. There is evidence that atherosclerosis starts in childhood and is progressive and that its severity correlates with the intensity of exposure to risk factors such as hypertension, dyslipidemia, obesity, diabetes, and smoking [3]. Furthermore, subclinical atherosclerosis in adolescents and young adults substantially increases the risk of overt CVD and of all-cause mortality later in life [4]. Besides atherosclerotic CVD, congenital heart diseases, rheumatic heart disease, cardiomyopathies, infective endocarditis, among others, are contributors to the burden of disease in adolescents and young adults.

### Cardiovascular health and disease in adolescents and young adults

A clear understanding of the epidemiology of CVD in adolescents and young adults is crucial for effective public health interventions to stem the growing burden of CVD in this young population and later in their lives. A recent study by Sun et al. published in *BMC Medicine* provides important information on the global, regional, and national burden of CVD among youths and young adults [5]. In this analysis of the Global Burden of Disease Study 2019, the authors calculated the age-standardized incidence, prevalence, DALYs, and mortality rate of CVD in individuals aged 15–39 years from 1990 to 2019, and proportional DALYs of CVD attributable to associated risk factors. They observed a decline in age-standardized (per 100,000 population) DALYs (1257.5 to 990.6) and mortality rate (19.8 to 15.1) for CVD from 1990 to 2019. However, the age-standardized (per 100,000 population) incidence rate (126.7 to 129.9) and prevalence rate of CVD (1477.5 to 1645.3) increased significantly in the same period [5]. The prevalence rate was highest in countries with low- and low-middle-sociodemographic index and the increase in this rate over time was faster compared to countries with high and high-middle sociodemographic index. Furthermore, high systolic blood pressure, high body mass index, and high low-density lipoprotein cholesterol were the three major contributors to DALYs for CVD globally, with household air pollution being an additional important contributor in countries with low and low-middle sociodemographic index [5].

**Fig. 1:**
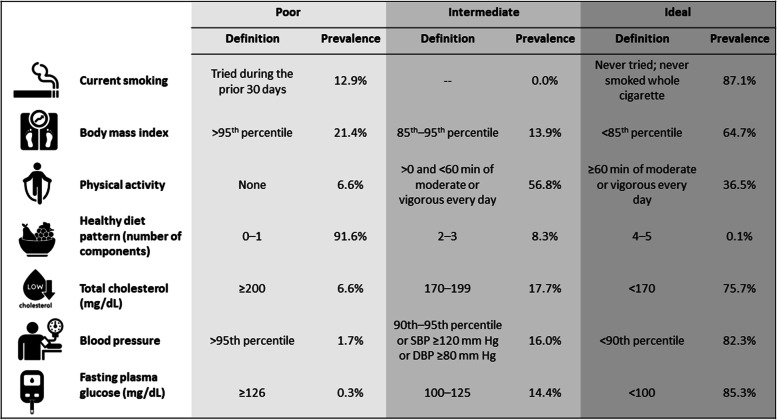
Definitions and prevalence of poor, intermediate, and ideal cardiovascular health metrics for adolescents ages 12–19 years according to the American Heart Association 2020 Strategic Goals (*definitions are taken from the American Heart Association 2020 Strategic Goals *[3]*, and unadjusted prevalence are based on the National Health and Nutrition Examination Survey 2009 to 2010 *[2])

These findings reiterate the importance of starting CVD prevention early in the life course. In fact, behaviors associated with cardiovascular health or risk of CVD in middle age and above are often established in childhood, adolescence, or young adulthood. For instance, initiation of smoking is commonly observed during adolescence, and dietary and physical activity patterns in adolescence frequently persist into adulthood [3]. How to define and evaluate cardiovascular health in children and adolescents is challenging. In 2010, the American Heart Association (AHA) introduced the concept of cardiovascular health, in recognition of the overwhelming body of evidence showing that individuals who reach middle age without traditional CVD risk factors have greater longevity, higher morbidity-free survival, greater health-related quality of life in older age, and substantially lower healthcare costs later in life [3]. This construct of cardiovascular health is defined using 7 health metrics, including 4 lifestyle factors (nonsmoking status, healthy diet, physical activity at goal levels, and normal weight) and 3 clinical factors (normal blood pressure, plasma glucose, and blood cholesterol levels) consistently shown in epidemiologic studies to be associated with healthy longevity [3]. Based on these metrics and with levels specific to children and adults, cardiovascular health is classified as ideal, intermediate, or ideal (Fig. 1). In the National Health and Nutrition Examination Survey (NHANES) 2009–2010, only half of US adolescents aged 12 to 19 years fulfilled five or more criteria for ideal cardiovascular health. More worrisome, 9 in 10 adolescents consumed a poor diet (Fig. 1) [2]. A high prevalence of cardiovascular risk factors is also observed in children and adolescents from low- and middle-income countries [6, 7].

### Cardiovascular disease prevention in adolescents and young adults

Several population-wide interventions for primordial prevention of CVD have shown positive results. These include statutory regulations that restrict unhealthy food and beverage marketing to children, increased taxations to limit the consumption of unhealthy highly processed food products, subsidies for healthier products such as fruits and vegetables and staple foods to support nutrition for those with lower incomes, supply-chain incentives to support the production of healthy foods, and nutrition labeling to help consumers make healthier dietary choices [7, 8]. Nutrition and physical activity initiatives in schools, along with parent-focused social marketing campaign, have also been shown to positively impact children and adolescent lifestyles [8]. Public health campaigns and interventions to prevent tobacco initiation and promote cessation should be reinforced. It is also important to tackle the new health challenges associated with the increasingly popular alternative tobacco products and e-cigarettes. To be successful, population-based interventions need to be tailored to the local context with a special attention given to their sustainability, they should be supported by strong multilevel leadership and policies, and their implementation and effectiveness should be continuously monitored and evaluated for strategic improvement [8]. A strong advocacy is particularly needed in low- and middle-income countries where the burden of communicable, maternal, and neonatal diseases is still high, and CVD is not yet considered a top public health priority.

The ongoing economic, technologic, and societal changes call for a constant review of and potential update of public health interventions. Digital technologies represent a major opportunity for health promotion among adolescents and young adults. With the global reach and integration of digital technologies in their lives, electronic health (eHealth) interventions have the potential to deliver CVD prevention interventions at a very large scale through smartphones, smartwatches, activity trackers, and tablets [9]. Because adolescents and young adults are generally in good health and have limited healthcare encounters, they may be less receptive to health promotion messages or less motivated to prioritize their long-term health. This issue could be partly overcome by their engagement in the creation and promotion of eHealth interventions and through youth advocacy.

As demonstrated by the analysis of the Global Burden of Disease 2019 [5], besides traditional cardiovascular risk factors such as high systolic blood pressure, high body mass index, and high low-density lipoprotein cholesterol, which are major drivers of the global burden of CVD, non-traditional cardiovascular risk factors such as household air pollution in low-income countries should not be overlooked [5]. Household air pollution is a major public health problem, particularly in sub-Saharan Africa where a large proportion of the population still relies on solid fuels for lighting, heating, and cooking [10]. More efforts are needed to raise population’s awareness on the health issues associated with household air pollution, to increase access to clean cooking solutions, and to promote improved home ventilation [10].

Finally, more research is needed to build the evidence base needed to support strategies to improve cardiovascular health in childhood, adolescence, and early adulthood. Emphasis should be placed on innovative interventions to promote healthy lifestyle behaviors, including interventions integrating digital technologies. Clinical trials of primary and secondary cardiovascular prevention are needed for young individuals with high cardiovascular risk, as they are usually excluded from these trials based on their age.

## Conclusions

Although improvements in acute cardiovascular care have translated into a decline in DALYs and deaths due to CVD in youths and young adults, especially in high-income countries, these gains have been offset by increasing trends of unhealthy lifestyle habits, physical inactivity, hypertension, overweight/obesity, dyslipidemia, and ultimately a rise in incident CVD. Adolescence and young adulthood are critical periods for building the foundation of cardiovascular health. Multilevel interventions to preserve ideal cardiovascular health tailored to this young population are needed to curb the burden of CVD. Investing in their health could generate a “triple benefit”: today, later in their lives, and for the next generation.

## Data Availability

Not applicable.
